# Retraction: Oxymatrine Synergistically Potentiates the Antitumor Effects of Cisplatin in Human Gastric Cancer Cells

**DOI:** 10.7150/jca.61392

**Published:** 2021-04-20

**Authors:** Yan Liu, Lei Qin, Tingting Bi, Wei Dai, Wei Liu, Quangen Gao, Genhai Shen

**Affiliations:** 1 Department of General Surgery, Wujiang No.1 People's Hospital affiliated to Nantong University, Suzhou, Jiangsu 215200, PR China; 2 Department of General Surgery, Hepatobiliary surgery, The First Affiliated Hospital of Soochow University, Suzhou, Jiangsu 215200, PR China

Regarding our paper 1, we recently found that during image preparation, one photograph of OMT treatment group in a different direction was accidentally misused in group OMT+CDDP in Figure 3a. And during the process of preparing Figure 7d, the selections of representative p-AKT and p-ERK images were mistakenly confused with the images from our other experiments. In order to maintain rigorousness and completeness of the article, the corresponding authors decide to withdraw this article and to perform further experiments to verify the data again. After careful consideration by all authors, we believe it is most appropriate to retract the article at this time. We sincerely apologize to readers, editors, and reviewers for our mistakes.
